# Bridging the Gap: Validating Mobile Telemedicine for Moyamoya Disease Diagnosis

**DOI:** 10.31662/jmaj.2024-0031

**Published:** 2024-04-01

**Authors:** George Kwok Chu Wong

**Affiliations:** 1 Division of Neurosurgery and Department of Surgery, Faculty of Medicine, The Chinese University of Hong Kong, Hong Kong, China

**Keywords:** Medicolegal, Moyamoya, Telemedicine

In the rapidly evolving landscape of medical technology, the efficacy of novel systems for providing accurate and reliable diagnoses is paramount. The article titled “The Efficacy of the Mobile Telemedicine System for Digital Subtraction Angiography of Moyamoya Disease Compared to Picture Archiving and Communication System” delves into the potential of mobile telemedicine in the context of Moyamoya disease diagnosis, particularly when compared with the established picture archiving and communication system (PACS) ^(1)^. This validation study holds immense importance not only for clinical applications but also for medico-legal purposes ^(2)^.

Mobile telemedicine has emerged as a promising avenue in healthcare, breaking down geographical barriers and providing access to medical expertise in real time. The study under discussion explores its application in digital subtraction angiography for the diagnosis of Moyamoya disease, a rare but serious condition affecting the blood vessels in the brain. By comparing its efficacy with that of conventional PACS, the research sheds light on the potential of mobile telemedicine to revolutionize diagnostic practices.

A validation study, conducted with rigorous methodology and statistical analysis, presents compelling evidence regarding the clinical validity and reliability of the mobile telemedicine system in angiographic diagnosis. The accuracy of digital subtraction angiography, a crucial imaging technique for this condition, is demonstrated through comprehensive comparisons with PACS. These findings provide confidence in the feasibility of incorporating mobile telemedicine into routine clinical practice.

Beyond the realm of clinical applications, the validation of mobile telemedicine gains significance in the medico-legal domain. Medico-legal considerations are central to ensuring the credibility of diagnostic tools and practices, especially when the stakes involve complex conditions like Moyamoya disease. The validation study serves as a cornerstone for establishing the credibility of mobile telemedicine systems, offering a solid foundation for legal acceptance of their use in diagnostic procedures.

Moyamoya disease often requires swift and accurate yet expert diagnosis for timely management decision. The mobile telemedicine system, as demonstrated in the study, has the potential to enhance accessibility to expert opinions, regardless of geographical constraints. This is particularly relevant in emergency situations where immediate consultation with specialists can be critical. By facilitating faster and more widespread access to diagnostic services, mobile telemedicine addresses a crucial aspect of healthcare delivery. In addition to accessibility, further study should investigate the potential cost efficiency of mobile telemedicine in angiographic diagnosis. The reduced need for physical presence in specialized medical facilities and the possibility of remote consultations may translate into economic benefits for healthcare providers and patients. This aspect, though not the primary focus of the study, adds to the overall appeal and feasibility of integrating mobile telemedicine into routine clinical practice.

In conclusion, the validation study on the efficacy of mobile telemedicine for Moyamoya disease diagnosis presents a compelling case for its integration into clinical practice. The findings not only emphasize its clinical validity and reliability but also underscore the importance of such validation studies for medico-legal purposes. As we navigate the evolving landscape of healthcare technologies, it is imperative to ensure that new systems meet the highest standards of accuracy and credibility, laying the groundwork for a future where mobile telemedicine plays a pivotal role in improving patient outcomes and streamlining healthcare delivery.

## Article Information

### Conflicts of Interest

None

### Disclaimer

George Kwok Chu Wong is one of the Editors of JMA Journal and on the journal’s Editorial Staff. He was not involved in the editorial evaluation or decision to accept this article for publication at all.
